# Resolution of Chronic Diarrhoea Following Treatment of Periodontal Disease in a Cat

**DOI:** 10.3390/ani16050759

**Published:** 2026-03-01

**Authors:** Samantha Taylor, Charlie Tewson, Victoria Edmondson

**Affiliations:** 1 Lumbry Park Veterinary Specialists, Selbourne Road, Alton GU34 3HL, UK; 2 Friendly Vets Ltd., Denholm TD9 8LN, UK

**Keywords:** dental disease, microbiome, gum–gut axis, inflammatory bowel disease

## Abstract

An association between periodontal disease and comorbid disorders, including gastrointestinal signs, has been reported in cats and investigated in humans, where a bidirectional relationship between oral and gastrointestinal microbiomes exists. A 5-year-old neutered female domestic shorthair cat was presented with a 2-year history of small-bowel diarrhoea. The cat’s appetite was reduced, and coat quality had deteriorated. On examination, the cat was found to have periodontal disease affecting multiple teeth and a matted coat. Biochemistry revealed mild hyperproteinaemia, haematology was unremarkable, cobalamin was normal, folate was elevated, and retroviral testing was negative. Abdominal ultrasound showed diffuse muscularis thickening without other abnormalities, and dental examination and radiography revealed missing teeth, a root remnant, stage 4 periodontal disease, and tooth resorption. Multiple extractions were performed, and multimodal analgesia was provided, including locoregional dental blocks. The cat’s appetite, body condition, energy levels, and coat quality improved after the procedure, and the diarrhoea completely resolved within a month of the procedure without any changes in diet, physical, social environment, or the use of any medications, and did not recur during the following 7 months. This case illustrates the potential role of periodontal disease in the development of gastrointestinal disease and the benefits outside the oral cavity of managing dental disease in cats.

## Simple Summary

Periodontal disease is a progressive infection and inflammation of the tissues supporting a cat’s teeth. It is common in cats of all ages and causes discomfort and tooth loss, can affect a cat’s appetite, and has been linked to other health problems. This case report describes a cat with severe periodontal disease and chronic small-bowel diarrhoea for 2 years despite dietary modification. The cat also had a matted coat and reduced appetite. After treatment of periodontal disease with extractions and dental hygiene, the diarrhoea completely resolved without any other changes or interventions. The cat gained weight and the coat improved. This case illustrates the potential connection between periodontal disease and gastrointestinal problems and emphasises the importance of the detection and prompt management of dental problems to improve feline health and welfare.

## 1. Introduction

Dental disease is common in cats [1], and periodontal disease is prevalent in this species and causes tooth loss and pain [2]. Causes include interactions between the host’s immune system and bacterial infections of the periodontium (gingiva, alveolar bone, periodontal ligament) resulting in inflammation and destruction of the tissues and hence eventual tooth loss [3]. Genetic predisposition and oral microbiome dysbiosis may play a role in the development of the condition [4]. In addition to gingivitis, gum recession, and oral pain, periodontal disease has been associated with systemic diseases in several species such as humans [5] and dogs [6]. Cats with periodontal disease had 1.79 times the odds of having a comorbidity than cats without periodontal disease in a recent study [2]. However, the impact of dental health on other organ systems is not well recognised in this species, yet it could be another motivator to treat poor dental health.

In people, a link between periodontal disease and gastrointestinal disease is a growing area of study, with inflammatory bowel disease [7], colorectal cancer [8], and ulcerative colitis [9] associated with gingival disease. The term ‘gum–gut’ (or oral–gut) axis has been coined to describe the bidirectional relationship between oral pathologies and the gastrointestinal tract [10,11]. Oral and intestinal dysbiosis and shared immunoinflammatory responses between the two areas have been described in people to result in inflammation and intestinal disease [11,12]. The oral microbiome of cats with periodontal disease differs from that of healthy cats [4,13], but the significance of this difference to the intestinal microbiome and intestinal inflammation is not fully understood.

This case study reports resolution of chronic small-bowel diarrhoea and weight loss following the treatment of severe periodontal disease in a cat without any other treatments (e.g., nutritional, antimicrobial, environmental), suggesting dental interventions could benefit gastrointestinal health and aiming to contribute to the literature supporting early diagnosis and treatment of dental disease in this species to improve both the health of the mouth and the health of organ systems outside the oral cavity.

## 2. Case Description

**Figure 1:**
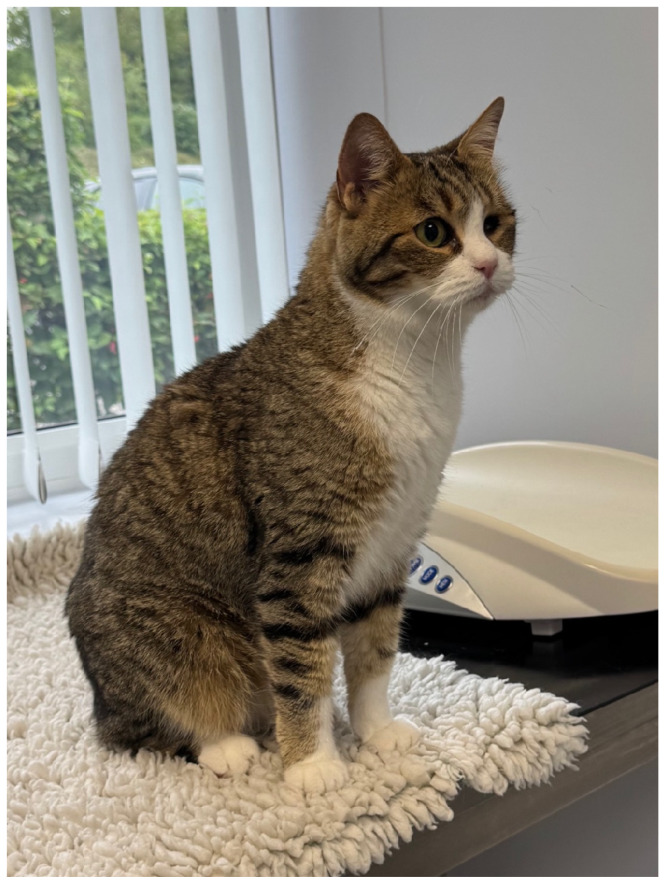
A 4-year-old female neutered domestic shorthair cat presenting with chronic diarrhoea and reduced coat quality.

A 5-year-old female neutered domestic shorthair cat (Figure 1) originally from Italy but resident in the UK for two years.

The cat was indoor only, lived with one other cat (sibling), previously vaccinated (herpesvirus, calicivirus, panleukopenia virus, feline leukaemia virus), and treated with endo- and ectoparasite prevention. The cat was fed a complete wet diet for cats with gastrointestinal disease (Gastrointestinal, Royal Canin, Castle Cary, England). When rescued as a kitten, the cat had severe ocular disease (assumed related to herpesvirus infection) leading to unilateral phthisis bulbi and has suffered chronic small-bowel diarrhoea consistently for the last two years, only partially improving when the diet was changed to the gastrointestinal food. The faeces were always cow-pat consistency (score 6–7 using the Purina faecal scoring chart) [14], passed once a day. Her appetite fluctuated and she was seen to chew preferentially on the left side of her mouth.

**Figure 2:**
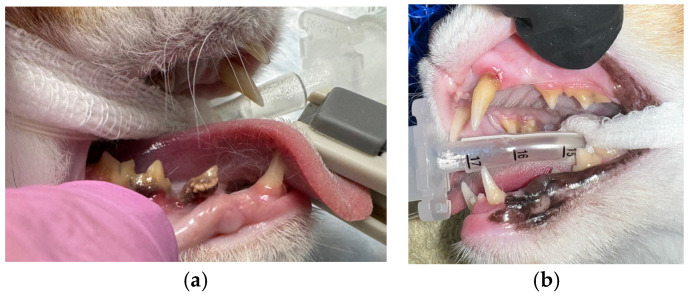
(**a**,**b**): Severe multifocal periodontal disease.

On physical examination, the cat was in slightly reduced body condition (3/9), 3.5 kg, bright and alert. The left eye was sunken into the socket but not inflamed or obviously painful. The cat’s coat quality was reduced, with some areas of matted fur and scale. Conscious dental examination revealed severe periodontal disease affecting multiple teeth (Figure 2a,b).

### 2.1. Investigation of Chronic Diarrhoea

**Figure 3:**
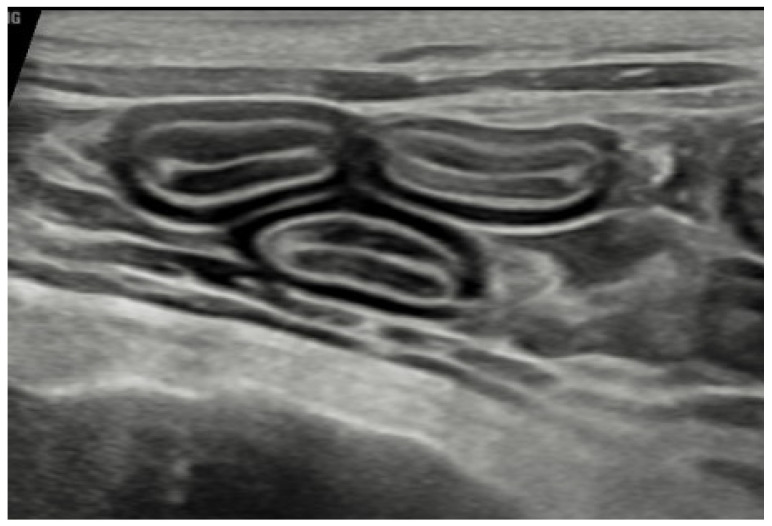
Ultrasound image of loops of jejunum showing mild muscularis thickening.

Retroviral testing in-clinic was negative. Haematology results were within normal limits, and biochemistry revealed no significant abnormalities except a mild increase in total protein (84 g/L, reference interval 56–81). To examine malabsorption, B12 and folate were measured; B12 was normal at >550 pmol/L (reference interval [RI] 220–500), and folate was elevated at 40.8 nmol/L (RI 19.0–37.0). Trypsin-like immunoreactivity was within the reference interval. Specialist abdominal ultrasound was performed to examine the gastrointestinal tract, other organs, and lymph nodes for indications of pathology. This revealed some mild generalised small-intestinal muscularis thickening (Figure 3) and mildly prominent mesenteric lymph nodes (too small to sample). No other abnormalities were detected, and all other organs were normal. Urinalysis was unremarkable. Next steps in investigating the chronic diarrhoea included faecal analysis and endoscopic or surgical intestinal biopsy; however, given the severity of the periodontal disease, dental treatment was prioritised with a plan to further investigate the diarrhoea once the cat recovered.

### 2.2. Dental Examination and Radiography

**Figure 4:**
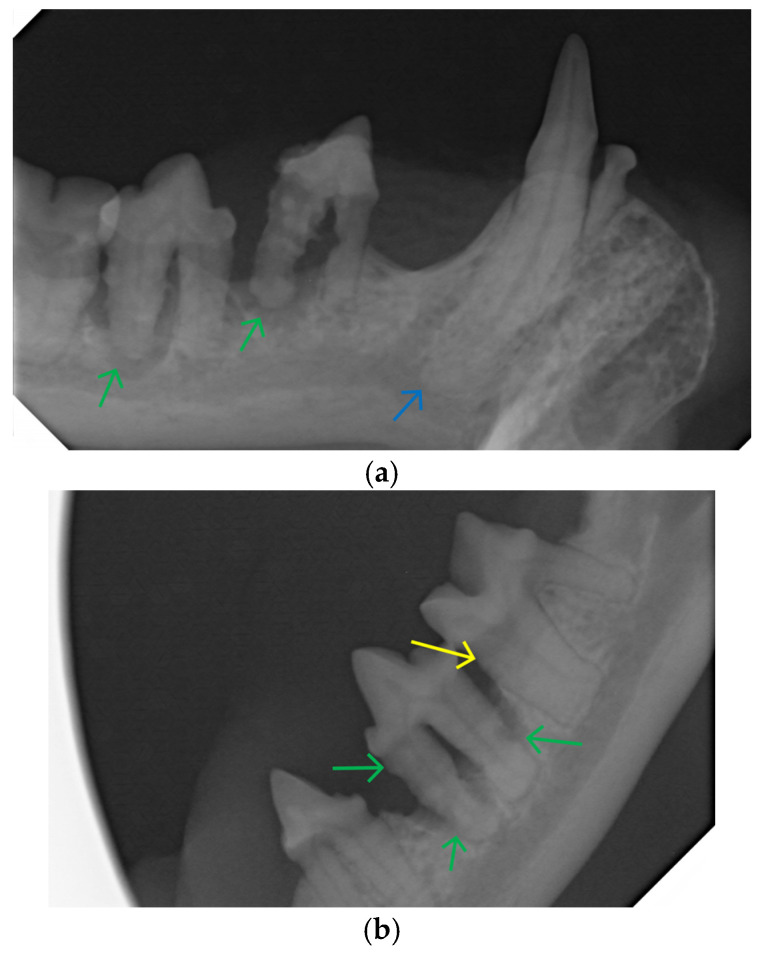

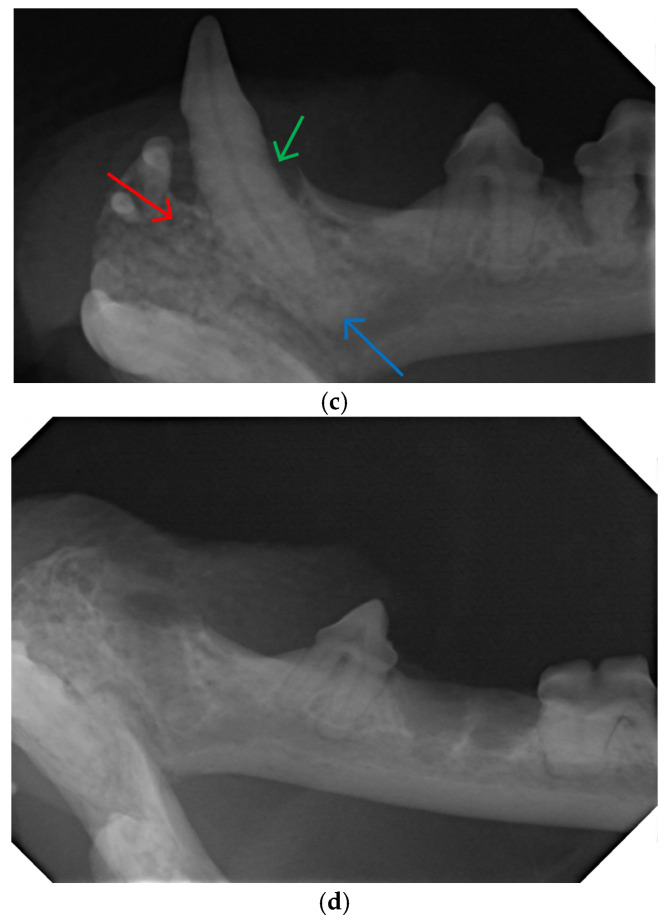
(**a**): Bisecting angle radiograph of the right rostral mandible with horizontal bone loss. The right mandibular third and fourth premolar teeth (407, 408) both demonstrate 100% attachment loss over the distal roots (green arrows). The 407 is significantly extruded from its normal position. The right mandibular canine tooth 404 shows a loss of definition of the periodontal ligament around the root apex (blue arrow). The surrounding mandibular bone has a mottled texture consistent with alveolar osteitis. (**b**): Parallel view radiograph of the left mandible. The left mandibular fourth premolar (308) demonstrates around 80% radiographic attachment loss (green arrows), and stage 3 (“through and through”) furcation exposure. The radiographic alveolar bone height on the mesial aspect of the left mandibular first molar (309) is reduced by around 25% (yellow arrow). (**c**): Bisecting angle radiograph of the left rostral mandible. The left mandibular canine tooth (304) shows significant attachment loss (green arrow), and a mottled radiopacity consistent with alveolar osteitis (red arrow). The root apex appears to be affected by type 2 tooth resorption as demonstrated by the loss of periodontal ligament space (blue arrow). (**d**): Post-extraction radiograph of the left rostral mandible demonstrating complete extraction of 303, 304 and 308.

Teeth are described with the modified Triadan System [15]. Significant findings included:

- Stage 4 (severe) periodontal disease: 108, 109, 204, 301, 303, 304, 308, 407, 408, 409 (Figure 4a–c)
- Stage 3 (moderate) periodontal disease: 309
- Retained root remnant: 202
- Missing teeth: 103, 106, 203, 206, 303, 401, 402
- Tooth resorption type 2: 304, 404 (Figure 4a,c)

### 2.3. Dental Treatment

This involved the surgical extraction of 108, 109, 204, 301, 303, 304, 308, 407, 408, 409, the root remnant of 202, and suturing of mucogingival flaps with 5-0 simple interrupted Monocryl. For the 304, despite the radiographic appearance of type 2 tooth resorption and a loss of periodontal ligament space, full extraction of the whole root apex was possible. Closed root planing was performed for the left mandibular first molar tooth (309). Tooth 404 (right mandibular canine) was affected by early type 2 radiographic tooth resorption but not periodontal disease. A recommendation was made for radiographic monitoring of this tooth.

Post-extraction radiographs were performed for of all extraction sites (Figure 4d).

### 2.4. Analgesia for Dental Procedures

- Methadone 0.3 mg/kg as part of pre-medication (with dexmedetomidine 5 mcg/kg and alfaxalone 0.5 mg/kg IM).
- Locoregional analgesia with maxillary and inferior alveolar nerve blocks using 0.5% bupivacaine, 0.15 mL at each site.
- Meloxicam 0.2 mg/kg SC given in recovery (fluid therapy provided during anaesthesia at 3 mL/kg/h with blood pressure monitoring).

Additionally, maropitant at 1 mg/kg IV was provided to reduce nausea and encourage post-operative food intake, and the cat was groomed to remove any matting that could cause discomfort in recovery.

As per current dental treatment guidelines, no antibiotics were prescribed pre-, peri-, or postoperatively [16].

### 2.5. Recovery and Case Outcome

The cat recovered rapidly from the procedure and was discharged that evening with ongoing meloxicam (Norbrook Laboratories Ltd, Newry, Northern Ireland) to be given 24 h later PO if eating and not vomiting along with transmucosal buprenorphine (Summit Pharmaceuticals, Kidlington, England) 0.02 mg/kg TID. The following day, the cat was bright and eating small volumes of normal wet diet frequently. The buprenorphine was not required, and only two doses of meloxicam were given. Further investigation of the chronic diarrhoea was planned to include endoscopy and faecal analysis.

**Figure 5:**
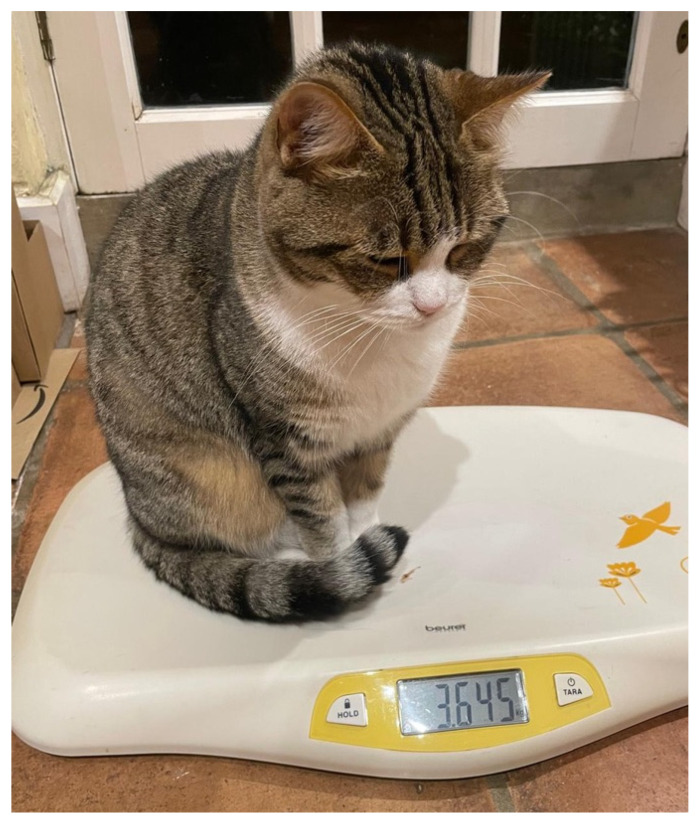
The cat 6 months after dental treatment showing 164 g weight gain, a shiny coat, and no matting.

Post-procedure, the cat showed a gradual consistent improvement in appetite, eating well consistently. Additionally, improvements in coat quality, demeanour, and energy levels were observed within 2 weeks of the procedure. After 1–2 weeks, a notable improvement in faecal consistency was observed (score 3–4). The diarrhoea completely resolved within one month (faecal score consistently 2–3) without other interventions. There was no diet change, no medications administered, or environmental adjustments made (physically or socially). Diarrhoea has not recurred in the seven-months post-dental procedure. The cat has gained 164 g in weight (Figure 5), and body condition score has increased to 4/9. The cat has a notable improvement in coat quality, with no visible matting and a shiny coat, and is described as much brighter and interactive than prior to the procedure.

## 3. Discussion

This case report describes a case of chronic small-intestinal diarrhoea that was unremitting for a 2-year period. The investigations excluded extraintestinal causes and identified evidence of chronic enteropathy as suggested by the chronicity and ultrasound findings of muscularis thickening. Further investigation was planned, but the diarrhoea completely resolved without any intervention beyond dental extractions and hygiene. Diet was not changed, antibiotics were not administered, and the environment and social interactions remained identical. It is therefore proposed that the case provides evidence that management of periodontal disease can result in improvements in systemic health as illustrated by weight gain and improved coat quality, but also that the ‘gum–gut’ axis may exist in this species, and treating periodontal pathology could improve chronic gastrointestinal signs. The cat had suffered diarrhoea for 2 years, which had not resolved with diet change. Investigations revealed mildly elevated folate, which has been hypothesised to reflect intestinal dysbiosis [17], although the significance of hyperfolataemia remains unclear. Muscularis thickening was identified on abdominal ultrasound, which could indicate underlying small-cell lymphoma or inflammatory bowel disease [18]. Further investigations such as endoscopy were planned, but treatment of the periodontal disease took priority. Given the dramatic and sustained response to dental treatment alone, further investigations were not pursued.

Associations between periodontal disease and systemic disease have been reported in people [5] and dogs [6,19], but this is less well studied in cats. O’Neill and others (2023) reported associations between periodontal disease and comorbidities with affected cats 1.79 times the odds of having at least one comorbid disorder [2]. That study showed haircoat disorder and diarrhoea to be significantly associated with periodontal disease (as hypothesised in this case) along with a number of other diverse conditions/clinical findings including cardiac arrythmias, chronic kidney disease, aural discharge, and hairballs. The authors suggested more evidence for the association could include dental intervention studies and monitoring of response to confirm the hypothesis of the connection illustrated in this single case report.

Transient bacteraemia is often assumed to be the mechanism for the association between oral and gastrointestinal disease, but other mechanisms have been studied in people, such as systemic immune stimulation causing a generalised inflammatory response [11], or pathogens and their metabolites spreading to other parts of the body [5,9]. In human medicine, recent studies showing links specifically between oral cavity disease and gastrointestinal diseases have been published and the ‘gum–gut (or oral–gut) axis’ proposed as a term to describe this bidirectional relationship [11]. Conditions such as inflammatory bowel disease have been linked to periodontal disease, with mechanisms such as dysbiosis and immune stimulation in the gut from bacteria in the saliva suggested [11], and even a link with gastrointestinal cancers discussed [8]. In cats, these specific associations are understudied, and this report did not analyse the cat’s oral or gastrointestinal microbiome pre- and post-dental treatment, which would help investigate this concept.

As a single case report, the association between the cat’s periodontal disease and diarrhoea cannot be confirmed. Other explanations for the resolution of diarrhoea in this case could be hypothesised, such as a reduction in pain and stress due to periodontal disease, as gastrointestinal signs can be a ‘sickness behaviour’ associated with challenging environments and individual cat temperaments [20], and it is difficult to definitively prove ‘cause and effect’ in such real-life case examples. However, the case report still contributes to the literature on the potential benefits of the management of periodontal disease in cats with gastrointestinal signs and may encourage practitioners to consider treating this aspect of a cat’s health prior to invasive and expensive additional investigations into chronic diarrhoea, if appropriate. As was planned in this case, if treatment of the oral cavity pathology fails to resolve the diarrhoea, then further work up is indicated.

Further research including larger numbers of cats with periodontal disease and other disorders assessed before and after treatment of dental disease will help describe these associations and add confidence to the association.

## 4. Conclusions

The present case report describes the resolution of chronic small-bowel diarrhoea, as well as improvement in general health, body condition, and well-being in a cat with severe periodontal disease. Although the prevalence of comorbid conditions with periodontal disease has been previously described, the impact of effective dental treatment on pre-existing and chronic diarrhoea with evidence of gastrointestinal wall abnormalities has not been reported. This case emphasises the need for dental examination in all cats presenting with ill health and prompt and thorough management of periodontal disease when present.

## Data Availability

All clinical data is included in the report.
